# Global gene expression during stringent response in *Corynebacterium glutamicum *in presence and absence of the *rel *gene encoding (p)ppGpp synthase

**DOI:** 10.1186/1471-2164-7-230

**Published:** 2006-09-08

**Authors:** Olaf Brockmann-Gretza, Jörn Kalinowski

**Affiliations:** 1 Institut für Genomforschung, Centrum für Biotechnologie, Universität Bielefeld, Universitätsstrasse 25, D-33615 Bielefeld, Germany

## Abstract

**Background:**

The stringent response is the initial reaction of microorganisms to nutritional stress. During stringent response the small nucleotides (p)ppGpp act as global regulators and reprogram bacterial transcription. In this work, the genetic network controlled by the stringent response was characterized in the amino acid-producing *Corynebacterium glutamicum*.

**Results:**

The transcriptome of a *C. glutamicum rel *gene deletion mutant, unable to synthesize (p)ppGpp and to induce the stringent response, was compared with that of its *rel*-proficient parent strain by microarray analysis. A total of 357 genes were found to be transcribed differentially in the *rel*-deficient mutant strain. In a second experiment, the stringent response was induced by addition of DL-serine hydroxamate (SHX) in early exponential growth phase. The time point of the maximal effect on transcription was determined by real-time RT-PCR using the histidine and serine biosynthetic genes. Transcription of all of these genes reached a maximum at 10 minutes after SHX addition. Microarray experiments were performed comparing the transcriptomes of SHX-induced cultures of the *rel*-proficient strain and the *rel *mutant. The differentially expressed genes were grouped into three classes. Class A comprises genes which are differentially regulated only in the presence of an intact *rel *gene. This class includes the non-essential sigma factor gene *sigB *which was upregulated and a large number of genes involved in nitrogen metabolism which were downregulated. Class B comprises genes which were differentially regulated in response to SHX in both strains, independent of the *rel *gene. A large number of genes encoding ribosomal proteins fall into this class, all being downregulated. Class C comprises genes which were differentially regulated in response to SHX only in the *rel *mutant. This class includes genes encoding putative stress proteins and global transcriptional regulators that might be responsible for the complex transcriptional patterns detected in the *rel *mutant when compared directly with its *rel*-proficient parent strain.

**Conclusion:**

In *C. glutamicum *the stringent response enfolds a fast answer to an induced amino acid starvation on the transcriptome level. It also showed some significant differences to the transcriptional reactions occuring in *Escherichia coli *and *Bacillus subtilis*. Notable are the *rel*-dependent regulation of the nitrogen metabolism genes and the *rel*-independent regulation of the genes encoding ribosomal proteins.

## Background

Bacteria cultured in a nutritionally poor environment use the stringent response [1] to survive starvation. The main effector molecule produced during the stringent response is the alarmone (p)ppGpp (guanosine-5'-diphosphate-3'-diphosphate and guanosine-5'-triphosphate-3'-diphosphate, respectively), which is synthesized in *Escherichia coli *by the *relA *and the *spoT *gene products. During amino acid starvation the ratio between charged and uncharged tRNAs decreases at the aminoacyl acceptor site (A-site) of the ribosome, which leads to a stop of protein synthesis. Uncharged tRNAs trigger a signal to the ribosome-bound RelA protein, which catalyzes the phosphorylation of GTP with ATP as donor [1,2]. The alarmone (p)ppGpp then binds to the β-subunit of the RNA polymerase [3], which leads to an activation or repression of a variety of genes, resulting in a global reprogramming of the cell to adapt to the new situation. The most prominent result is a growth arrest, accompanied by the downregulation of genes that are strongly expressed in fast growing cells e.g. those encoding ribosomal proteins, cell division and DNA replication proteins as well as a reduction of the amount of stable RNA [4,5].

In our previous studies it was shown that in contrast to *E. coli*, *C. glutamicum *possesses a single bifunctional guanosine pentaphosphate synthetase, termed Rel [6]. A *rel *gene deletion mutant strain *C. glutamicum *RES167Δ*rel *was created and shown to be unable to synthesize (p)ppGpp after inducing an artificial amino acid starvation by DL-serine hydroxamate (SHX) [6]. SHX is a serine analogue, which competitively binds to the seryl-tRNA synthetase and thus prevents the seryl-tRNA from being charged [7]. This results in the induction of the stringent response by (p)ppGpp, newly synthesized through the action of the Rel protein in the wild-type strain. Furthermore, the *rel *mutant failed to downregulate ribosomal RNA transcription under stringent conditions and showed a growth requirement for the amino acids histidine and serine [5]. This observation proposes that transcription of at least parts of both biosynthesis pathways are under a positive stringent control. We had further investigated additional ribosomal genes involved into the stringent response, namely *rplK*, which is required for activating the Rel protein and is thus part of the mechanism to induce the stringent response of *C. glutamicum *[8].

In this study we continued our previous work to explore the regulatory network of the stringent response by comparing the *C. glutamicum *RES167 and *C. glutamicum *RES167Δ*rel *transcriptomes by using whole-genome microarrays. In a first approach, the transcriptomes were compared between exponentially growing cultures in complex growth medium indicating a complex pattern of differences in gene expression. In a second approach, the stringent response was induced by the addition of SHX. Therefore, an optimal time-point for harvesting total RNA was determined by a time-resolved characterization of the mRNA profiles of histidine and serine biosynthesis genes. Then, the transcriptomes of the *rel*-proficient and the *rel*-deficient strain were compared before and after addition of SHX in order to determine those genes whose transcription was directly affected by (p)ppGpp.

## Results

### Global gene expression in the exponential growth phase is highly different in a C. glutamicum *rel*-proficient strain and a *rel*-deficient mutant unable to produce (p)ppGpp

To study the effects of the effector molecule (p)ppGpp on a genome-wide scale the transcriptomes of a *rel*-proficient and a *rel*-deficient strain were compared by hybridization analysis using whole-genome DNA microarrays of *C. glutamicum *[9]. Therefore, both strains were grown in liquid LB complex medium and cell samples were taken at an O.D._600 _of 0.2. RNA isolation from *C. glutamicum*, labeling of probes and DNA microarray hybridization, including statistical analysis, were performed as described recently [9]. The spot intensities were measured and compared between RES167Δ*rel *(int_rel_) and RES167 as control (int_cont_). Each experiment was performed four times, using two biological and two technical replicates including a dye-swap. In accordance with the previously determined technical variation of the *C. glutamicum *DNA microarray employed, only genes with ratios of int_rel_/int_cont _greater than 2^0.6 ^or less then 2^-0.6 ^were considered as being differentially expressed. For these ratios the significance value was determined as 0.95, meaning a 95% chance that the gene is differentially regulated. In addition, a Students *t*-test was applied to filter the data set and only data with an error probability of p = 0.05 or better were used.

**Figure 1:**
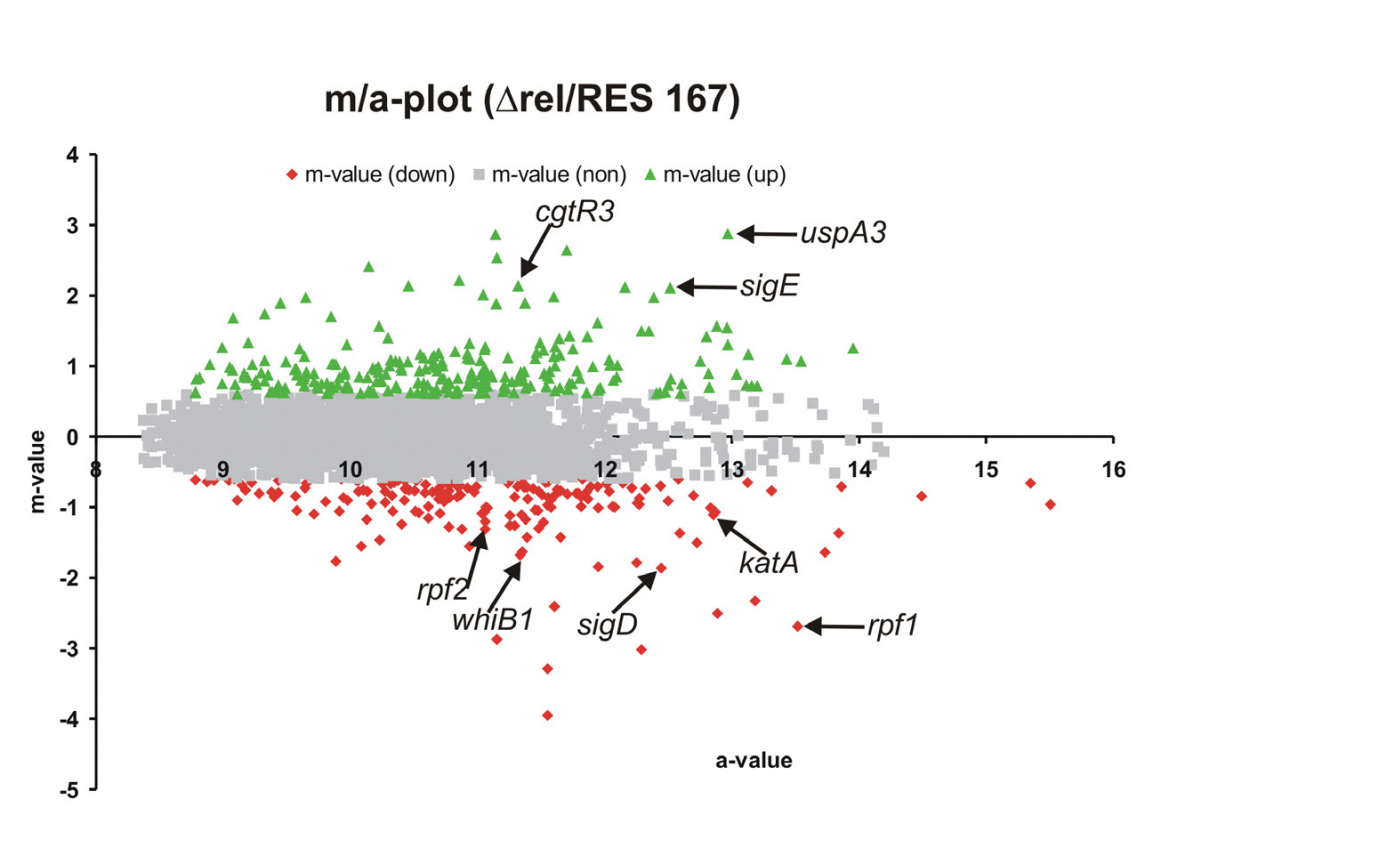
The m/a scatter plot comparing the transcriptomes of the *C. glutamicum rel*-deficient and the *rel*-proficient strains. Genes with a reduced transcription are indicated by red dots and those with an upregulated transcription by green dots. Genes described in the text and showing the strongest changes in the expression ratio are marked by arrows.

Figure 1 shows the results of this experiment depicted in a scatter plot. A total of 357 genes was found to be differentially expressed in the Δ*rel *mutant, of which 217 were transcribed stronger and 140 weaker than in the *rel*-proficient strain. Among the most prominent induced genes are *uspA3 *(putative universal stress protein), and the *sigE *gene (sigma factor of the extra-cytoplasmic function (ECF) family). The genes *sigD *(encoding another ECF-type sigma factor) as well as *rpf1 *and *rpf2 *(encoding proteins involved in bacterial cell-cell communication, [10]) are among the genes with the most strongly reduced expression.

Since global gene expression patterns were very different in the *rel-*proficient and the *rel*-deficient strain, and sigma factors responsible for global gene regulation are also affected, it was difficult to dissect primary effects of the *rel *mutation from secondary effects caused by differentially expressed regulatory genes. In order to overcome these problems, a second experimental strategy was employed to describe *rel*-dependent and *rel*-independent effects on global gene expression in which the stringent response was induced by the chemical DL-serine hydroxamate (SHX). The strategy consisted of two steps. First, the transcriptional effects of SHX addition on gene expression of stringently controlled genes were followed in a time course to determine the time point at which a maximal first order effect is exerted on gene expression. Then, microarray hybridizations were performed comparing the transcriptomes of both strains before and after addition of SHX for the dissection of *rel*-dependent and *rel*-independent effects during stringent response.

### The mRNA levels of serine and histidine biosynthesis genes in *rel*-proficient C. glutamicum cultures treated with serine hydroxamate are induced and reach a maximum ten minutes after induction of the stringent response

**Figure 2:**
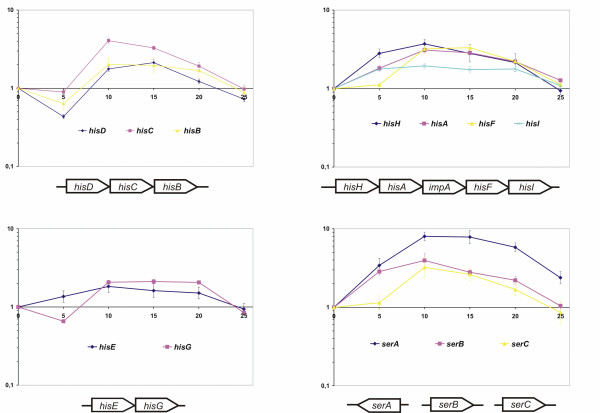
Time course analysis of expression of the histidine and serine biosynthesis genes after treatment with DL-serine hydroxamate. The samples were taken in five minute intervals up to a total of 25 minutes. The relative amount of mRNA was measured by real-time RT-PCR with a LightCycler instrument. The expression ratios were determined by calculating the ratio of t_0 _(untreated) and t_5–25 _(treated) samples from four experiments. Below the diagrams, the organization of the respective genes in the *C. glutamicum *chromosome is depicted.

In previous experiments, it was demonstrated that the *C. glutamicum *RES167Δ*rel *mutant grows poorly in liquid minimal medium without the supplementation of histidine and serine [5]. Apparently, the biosynthesis pathways of these amino acids are under control by a positive stringent response mediated by the Rel protein. In order to analyze transcriptional regulation of all genes involved in histidine and serine biosynthesis and to assess the timing of the effects of the stringent response on gene expression, sensitive real-time RT-PCR analyses on all serine and histidine biosynthesis genes were carried out in the *rel*-proficient *C. glutamicum *RES167 strain. Therefore cultures of *C. glutamicum*, growing exponentially in liquid LBG medium, were treated with 50 mM SHX. Samples were taken before addition of SHX (t_0_) and in five minute intervals up to a total of 25 minutes (t_5 _to t_25_). Total RNA was isolated from these samples and mRNA levels of the nine known histidine biosynthesis genes *hisED, hisDCB *and *hisHAFI *and the three serine biosynthesis genes *serA*, *serB *and *serC *was determined. For each time point after treatment with SHX, the relative mRNA level compared to the t_0 _value was calculated (Figure 2).

The results clearly showed an increase of mRNA levels of some of the tested genes already five minutes (t_5_) after SHX addition, with all being increased and with a maximum of induction after ten minutes (t_10_). At this time, mRNA levels of the histidine genes are increased twofold to threefold and serine genes were transcribed stronger by factors of 3 to 7.8. In later stages relative mRNA levels slowly decreased until they reached the initial values at around 25 minutes (t_25_). It is apparent that the histidine genes, which are localized on the chromosome in operon-like structures, are transcribed in similar patterns, supporting the idea that these genes are organized in the three operons *hisEG*, *hisDCB *and *hisHA-impA-hisFI*. The strongest induction was observed at the *hisC *and *hisH *genes (Figure 2). Among the serine biosynthesis genes, *serA *coding for D-3-phosphoglycerate dehydrogenase, the first enzyme of the serine biosynthesis, was the gene whose mRNA level was induced most strongly.

These elevated mRNA levels after addition of SHX were not detected in the Rel-deficient strain *C. glutamicum *RES167Δ*rel *(data not shown), supporting the interpretation that this is the cause for its requirement of histidine and serine as culture supplements to achieve normal growth [5].

### Comparison of transcriptional profiles of C. glutamicum *rel*-proficient and *rel*-deficient mutant strains before and after induction of the stringent response allowed to identify *rel*-dependent genes

To determine the genes directly affected by (p)ppGpp in *C. glutamicum*, exponentially growing cultures of the *rel*-proficient strain *C. glutamicum *RES167 and the *rel*-deficient mutant strain RES167Δ*rel *carrying a deletion in the (p)ppGpp synthase gene were treated with SHX. The comparison of global gene expression patterns in the *rel*-proficient and the *rel*-deficient mutant strain at inducing and non-inducing conditions should allow to define the regulatory networks involved in the stringent response. In addition, comparison of the data sets enabled the identification of those genes that are controlled by the action of the Rel protein (Rel-dependent stringent control) and of those which are not dependent on the action of the Rel protein (Rel-independent control).

Two *C. glutamicum *cultures were grown in liquid LBG medium up to an O.D._600 _of 0.2, at which SHX was added to a final concentration of 50 mM. For each strain, untreated samples (t_0_) and treated samples were taken ten minutes after SHX addition (t_10_) and the transcriptomes were compared as described above.

**Figure 3:**
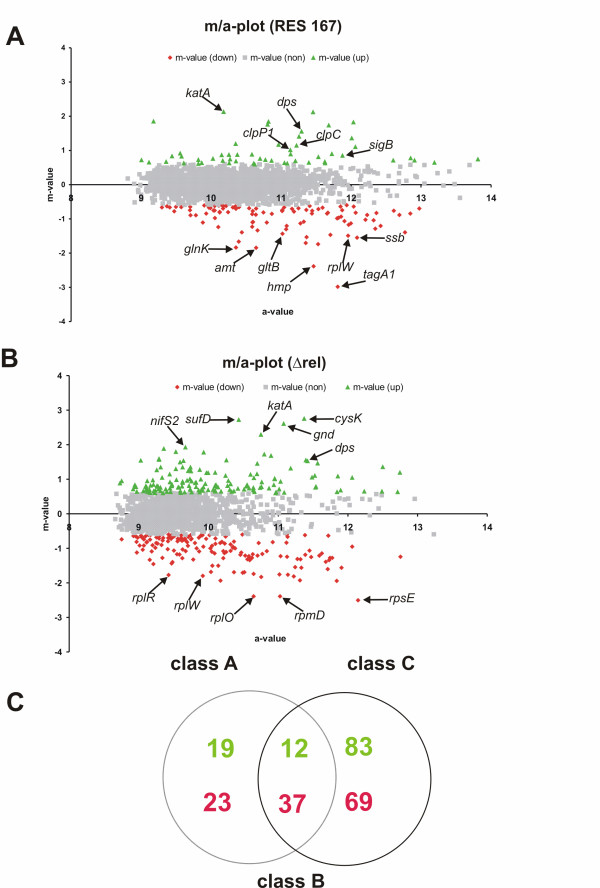
DNA microarray hybridizations of the *C. glutamicum *RES167 and the *C. glutamicum *RES167Δ*rel *mutant strain. **(A) **and **(B) **Scatter plots (m/a plots) of the experiments. Shown are all genes whose transcription has been significantly up- (green) or downregulated (red) after induction of the stringent response by DL-serine hydroxamate. Genes which showed the most prominent changes in expression are labelled. **(C) **Venn diagram of the differentially expressed genes in the *C. glutamicum *RES 167 and Δ*rel *mutant after treatment with SHX is shown. The numbers of genes which were placed in each of the three classes A, B and C is given. Green numbers represent upregulated genes, red ones represent downregulated genes.

In Figure 3 the results of the microarray experiments are shown in ratio/intensity (m/a) plots for both the *rel*-proficient and the *rel*-deficient strain. In the *C. glutamicum rel*-proficient strain transcription of 60 genes was found to be downregulated and of 31 genes to be upregulated under inducing conditions. The most prominent upregulated transcription was detected in genes which are involved into stress response, among them *katA *(catalase) and *dps *(DNA protection during starvation protein). The genes with the strongest downregulation are the *tagA1 *gene (DNA-3-methyladenine glycosylase) involved in DNA repair or belonging to nitrogen metabolism including *hmp *(putative NO-detoxification flavohemoprotein), *glnK *(nitrogen regulatory protein PII), *gltB *(glutamate synthase large subunit) and *amt *(ammonia transporter).

In the m/a plot of the *rel*-deficient strain (Figure 3B) it is shown that the genes with the strongest upregulation are again *katA*, *gnd *(6-phosphogluconate dehydrogenase) and thiol stress-related genes. Among the latter, the *sufD *and *nifS2 *genes are involved in the repair of damaged iron-sulfur clusters in proteins whereas *cysK *encodes cysteine synthase. The strongest downregulation is observed for genes encoding ribosomal proteins (e.g. *rpsE*, *rplO*, *rpmD*).

The data obtained on the differentially regulated genes were grouped into three classes: class A genes are under Rel-dependent stringent control and were found to be differentially expressed only when the untreated and SHX-treated cultures of the *rel*-proficient RES167 strain were compared and therefore are regulated by (p)ppGpp. Class B genes are under positive or negative *rel*-independent control and show a change in expression in RES167 and its *rel*-deficient derivative RES167Δ*rel*. In class C those genes were placed which showed a change in expression only when comparing the untreated and the SHX-treated *rel*-deficient mutant strain. By this classification scheme, the total number of 243 genes found to be differentially expressed in one or both experiments were ordered as follows: 42 genes in class A, 49 in class B and 152 genes in class C (Figure 3C). For reasons of clarity only the classes A and B are presented here in the form of tables (Table 1, 2) and, within the tables only genes which are annotated at least in the form of a COG classification (Clusters of Orthologous Groups of proteins, [11]) are listed. A full list is available as supplementary Table S1 [see Additional file 1].

### Class A genes are under (p)ppGpp mediated stringent control as they were found to be regulated SHX-treatment only in the *rel*-proficient RES167 strain but not in its *rel*-deficient derivative RES167Δrel

**Figure 4:**
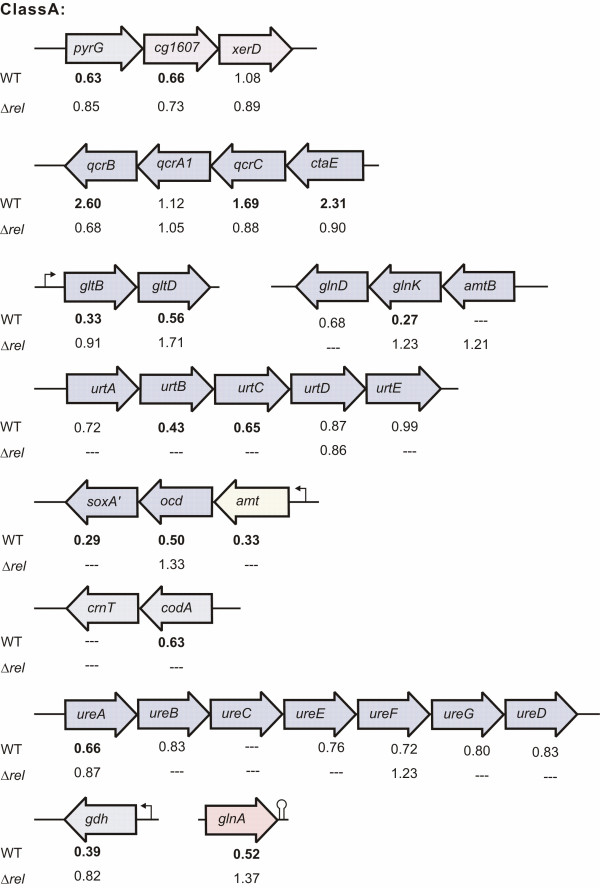
Graphical representation of selected genes and putative operons comprising genes of classe A in their genomic organization. Below each gene, the induction or repression ratios are given for both strains, *C. glutamicum *RES167 and its derived Δ*rel *mutant. Bold numbers indicate a significant induction or repression of the corresponding gene according to the filtering criteria (5% error probability) applied on the microarray data. Genes are not drawn to scale. Known transcription start points are marked by small arrows. Predicted rho-independent terminators are indicted by hairpins.

The hallmark of the stringent response is the downregulation of transcription of the genes encoding ribosomal RNA in a (p)ppGpp-dependent manner. In the microarray experiments, a number of genes displayed a similar change in expression dependent on the presence of the alarmone (p)ppGpp synthesized by the Rel protein. These genes can be grouped to functional complexes according to their annotation and the COG classification scheme (Table 1). As the dominant complex, nitrogen metabolism is apparently affected in *C. glutamicum *in a negative, Rel-dependent fashion. In particular the transcription of genes belonging to the AmtR repressor regulon [12] were found to be downregulated (*codA, gdh, glnA*, *glnK*, *gltB, amt-ocd-soxA, ureA, urtB-urtC*) (Table 1). In addition, two other genes encoding oxidoreductases (*betB*, *cg3370 *; COG class C) are found to be repressed in the presence of (p)ppGpp. Others are three genes encoding ribosomal proteins (*rpmG*, *rpsF*, *rpsS*) and one gene cluster involved in nucleotide biosynthesis and DNA repair (*pyrG*-*cg1607*, Figure 4).

The group of genes the transcription of which is positively affected in a Rel-dependent manner also comprises those encoding proteins involved in redox processes (*qcrB*-*qcrC-qcrA1-ctaE*, Figure 4). Other genes are involved in carbon metabolism (*ptsF*, *pyk*, *ldh*) or regulatory processes on protein (*ptpA*, *clpP1, clpC*) or mRNA level (*lexA*, *sigB*). Especially *sigB *is interesting since it encodes the alternative sigma factor (σ^B^) in *C. glutamicum *which is involved in global gene regulation at the transition from exponential growth to the stationary phase and in the general stress response [13,14].

### Class B genes are regulated Rel-independently and show a change in their expression in the *rel*-proficient strain and the *rel*-deficient mutant

**Figure 5:**
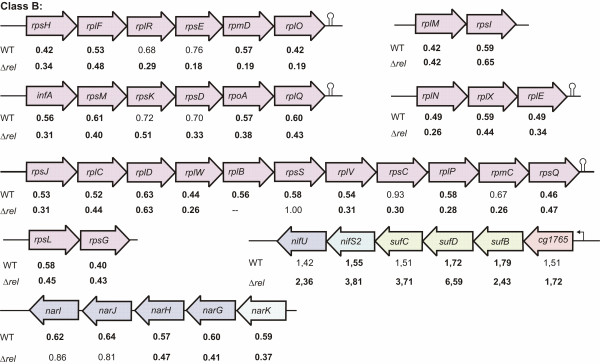
Illustration of putative operons of class B. For each experiment with the *C. glutamicum *RES 167 and its derived Δ*rel*-mutant the induction or repression ratios are given. Bold numbers indicate a significant induction or repression of the corresponding gene according to the filtering criteria (5% error probability) applied on the microarray data. Genes are not drawn to scale. Known transcription start points are marked by small arrows. Predicted rho-independent terminators are indicted by hairpins.

The most important members of this class that are regulated in a negative Rel-independent manner are genes coding for ribosomal proteins (COG class J: translation), of which 26 were found to be downregulated under inducing conditions (Table 2, Figure 5). Additionally, transcription of one other gene from the translational apparatus (*infA*) and of three genes involved in transcription or DNA replication (COG classes K and L) was repressed in both strains. As a further functional complex, the transcription of the putative nitrate reductase and transport genes (*narK-narG-narH-narJ-narI*) was found repressed.

In a Rel-independent way, transcription of genes involved in oxidative stress response (*katA *encoding catalase, *dps *encoding a DNA protection protein) was upregulated not only in the *rel*-proficient, but also in the *rel*-deficient mutant strain of *C. glutamicum *(Table 2). Additionally, the transcription of genes from the *suf/nif *gene cluster (*sufB-sufD-nifS2*) involved in the repair of Fe-S clusters damaged by oxidation [15] was induced under these conditions (Figure 5).

### Class C genes belong to a variety of functional complexes and show differential regulation only in the *rel*-deficient mutant

A large number of genes were found to be regulated after treatment with SHX only in the *rel*-deficient mutant but not in the *rel*-proficient strain (class C). Within this class additional genes belonging to the translational (COG class J) and transcriptional apparatus (COG class K) were grouped, in total, 18 genes encoding ribosomal proteins, *infB *encoding translation initiation factor 2 and *prfB *(peptide chain release factor 2), as well as *rpoB *(β subunit of RNA polymerase) as well as *cg2177*-*nusA *encoding proteins involved in transcription termination. As illustrated in an example (Figure 5) the transcription ratios of several ribosomal genes in the *rel*-proficient strain experiment are just below the significance value. Taken into account that most of these genes are organized in operons, it can be assumed they rather belong to class B than to class C.

The expression of genes involved in oxidative stress response has been found to be upregulated only in the *rel*-deficient mutant (*sod *encoding superoxide dismutase, *cg2674 *encoding a putative alkyl hydroperoxidase and *tpx *encoding a thiol peroxidase). Especially thiol oxidation stress is apparent since also the expression of the *fpr2-cysI-cysH-cysD *gene cluster for assimilatory sulfate reduction [16] as well as *cysK *encoding cysteine synthase are enhanced.

It is apparent that a number of transcriptional regulatory genes fall into class C. Among these are *cg1792 *encoding a WhiA homologue, *whiB2 *and *whiB4*, *cg0725 *encoding a MarR-family regulator, *clgR *encoding a stress-induced proteolysis regulator [17], *phoU *encoding the putative phosphate uptake regulator, and *cg3303 *encoding a regulator of the PadR family. All these genes were found to be repressed under stringent conditions in the *rel *mutant. Another group of genes encoding transcriptional regulators is found to be expressed stronger under these conditions, namely *cg1631 *encoding a MerR family regulator, *dtxR *and *fur*, encoding regulators implicated in metal uptake and homeostasis, *cg2500 *encoding an ArsR-type regulator, *cg2614 *encoding a TetR-type regulator, and *cg3315 *encoding a regulator of the MarR family [18,19]. This is indicative that cellular metabolism is not well balanced in the mutant but on the other hand this deregulation might cause secondary effects on global gene expression patterns not directly associated with the stringent response.

## Discussion

In earlier studies, we demonstrated that (p)ppGpp is the signalling molecule of the stringent response in *C. glutamicum *[6]. It is synthesized after inhibiting the seryl-tRNA synthetase by addition of DL-serine hydroxamate (SHX) to the growth medium. We also showed that the Rel protein is responsible for (p)ppGpp formation and that its presence is required for the normal function of the serine and histidine biosynthesis pathways [5].

In this study, we investigated the timing of the stringent response at the histidine and serine biosynthesis genes by real-time RT-PCR and the global effect of the stringent response on gene expression by using comparative DNA microarray hybridizations. In the following, the observations made will be discussed and the findings in *C. glutamicum *will be compared to the existing knowledge obtained in the model organisms *E. coli *and *B. subtilis*. However, these comparisons have inherent problems. The stringent response of *E. coli *has up to now not been assessed on a global scale in a mutant unable to synthesize (p)ppGpp and therefore no data exist on a probable *relA*/*spoT*-independent stringent response in this organism. In *B. subtilis *such a study has been carried out [20] although there the stringent response was induced by DL-norvaline in a mutant auxotrophic for the amino acids lysine and tryptophan.

As an initial microarray experiment comparing the transcriptomes of a *C. glutamicum rel *mutant strain with its *rel*-proficient parent yielded an extremely complex pattern of gene expression, the effects of the *rel *gene on stringent control of transcription were assessed by a second approach including the addition of SHX. In order to determine the optimal harvesting point, the genes encoding the enzymes of the histidine and serine biosynthetic pathways were used for establishing a time course of the stringent response on transcription by sensitive real-time RT-PCR. The induction of transcription of these genes by the stringent response was detected already five minutes after addition of SHX to the medium. The maximum induction of expression was measured 10 minutes after SHX addition. The strongest increase in expression was found in the case of the *hisC*, *hisH *and *serA *genes. Later, expression declined and the transcription ratios were back to normal levels after 25 minutes, indicating that the stringent response is completed at that point and the cells can resume normal growth again. In *S. typhimurium*, it was shown that an increase of the (p)ppGpp level was detectable immediately after SHX addition and reached its maximum after 7 minutes [21]. These results are in accordance with our experiments and show a rapid response of the cell to an artificially induced amino acid starvation. The histidine genes were among the first ones to be found under positive stringent control [1,22,23] and the effect of (p)ppGpp on the *his *operon in *E. coli *and *S. typhimurium *has been studied intensively [21,23] and was later confirmed by microarray experiments [24]. Our results are in accordance with the *E. coli *data concerning the induction of the histidine biosynthesis genes, but no induction of histidine or serine gene expression was observed in *B. subtilis *[20]. However, the exact mechanism of induction of *his *operon expression in *E. coli *is still unknown. The strong induction of the serine biosynthesis genes in *C. glutamicum *is unexpected since mainly histidine and arginine biosynthesis genes are reported to be regulated by the stringent response in *E. coli *and *B. subtilis *[1,20,25].

In order to identify stringently controlled genes as precise as possible, a filtering of the genes appearing differentially regulated in the microarray experiments was performed and three classes were established. The genes of Class A are differentially expressed only in the presence of a functional *rel *gene, and therefore, in the presence of (p)ppGpp. Dominant in this class are genes involved in nitrogen metabolism. Namely these are *amt *(encoding ammonia permease; [26]), *codA *(creatinine deaminase; [27]), *ureA *(urease γ -subunit; [28,29]), *gltB *(glutamine 2-oxoglutarate aminotransferase large subunit; [30]), *glnA *(glutamine synthetase; [31]), *glnK *(nitrogen regulatory protein PII; [32]), *urtBC *([33]) and *gdh *(glutamate dehydrogenase; [34]). In *E. coli*, an increased transcription of the *glnK *gene was observed under stringent conditions [24], probably reflecting the fact that *E. coli *has nitrogen sensing and signal transduction mechanisms quite different to those of *C. glutamicum *[35]. In this context it is interesting to note that the genes of nitrogen metabolism detected here were shown to be controlled by the transcriptional regulator AmtR in *C. glutamicum *[12,36]. It was demonstrated that the transcription of the genes of the AmtR regulon is upregulated under nitrogen limitation [37]. There are two different possible explanations for the observation that the genes of the AmtR regulon are under Rel-dependent stringent control. The first possibility is that AmtR is directly affected by (p)ppGpp in a sense that it strengthens its repressor function. Second, it might be speculated that especially the promoters of genes of the AmtR regulon are sensitive to (p)ppGpp.

Also the expression of the alternative sigma factor σ^B^, the supposed functional analogue of the *E. coli *σ^S ^[14], is induced after invoking the stringent response by an artificial amino acid starvation. In *E. coli*, σ^S ^controls the general stress response and the entry into the stationary phase of growth [38,39] and thus plays an important role in adaptation to nutritional and environmental stresses. In *E. coli*, σ^S ^expression is increased during the stringent response [24,39] but contrary to this the *B. subtilis *functional analogue σ^B ^seems to be involved only in general stress response and is not induced by the stringent response [20,40-42]. This leads to the assumption that a mechanism similar to *E. coli *is present in *C. glutamicum*, inducing the stress response as well as the entry into the stationary phase due to nutrient limitation. Here, an inherent problem of such analyses becomes obvious, namely how to distingush between a direct, Rel-dependent induction or repression of gene transcription and σ factor-mediated transcriptional changes. We tried to minimize this problem by analysing a relatively early time-point after induction as it was done for *E. coli *[24].

Another important cellular function is provided by the genes coding for subunits of the Clp protease (*clpP1*, *clpC*) that are under Rel-dependent, positive stringent control. This shows a strong parallel to results obtained in *Streptococcus pyogenes*, where the transcription of these genes was found to be upregulated in a Rel-independent manner upon an artificially induced amino acid starvation [43]. As published recently, the *clpP1P2 *and *clpC *genes of *C. glutamicum *are induced upon heat stress [17]. Proteases together with chaperones are the cells quality management system and involved in the regulation of transcriptional regulator genes [44]. It has been shown for *E. coli *that some proteins are rapidly degraded in an ATP-dependent manner during the stationary phase [45]. Also by means of the ClpP protease, *E. coli *adapts to the stationary phase on the proteomic level [46]. Thus it might be speculated that by inducing these genes during the stringent response a global change in gene expression is mediated by the changed availability of several regulatory proteins due to proteolysis.

It has to be noted that in the microarray analysis, the histidine and serine biosynthesis genes, which were found to be transcribed stronger in response to the induction of the stringent response in the *rel*-proficient strain, did not show up with ratios indicating 95% chance of differential regulation. This is explained by the much higher sensitivity of the RT-PCR approach which in turn means that a number of other genes whose transcription is influenced might have escaped this analysis and the stringent data filtering.

The most important complex of class B being upregulated Rel-independently is made of genes involved into the oxidative stress response. Namely these are the genes coding for catalase (*katA*) and the DNA protection during starvation protein (*dps*). These findings are in accordance with recent findings in *E. coli*. Dukan and Nyström [47] found that at the onset of the stationary phase several genes involved in a response to oxidative stress are induced. In this context the regulation of the *dps *gene is also of importance. It was shown recently that Dps proteins protect stationary phase cells against a variety of stresses, including oxidative stress [48] and it was also found to be upregulated during the stringent response in *E. coli *[24].

The most prominent members of genes with reduced expression are those for ribosomal proteins which form the dominant complex in this group. In *E. coli *and *B. subtilis*, the genes for ribosomal proteins showed a reduced expression after inducing the stringent response [20,24]. In contrast to *B. subtilis *[20,49] the ribosomal genes are Rel-independently controlled in *C. glutamicum*. This is an apparent discrepancy to the established model which interprets this decrease in expression by the reduced amount of ribosomal RNA available under stringent conditions and the subsequent transcriptional repression on ribosomal protein operons by ribosomal proteins. Since in *C. glutamicum*, repression of many genes encoding ribosomal proteins is found in both the *rel*-proficient and the *rel*-deficient strain after addition of SHX, it might be explained by empty tRNAs which cause a stall in protein biosynthesis by the ribosomes leading to the disassembly of ribosomes. This in turn would cause a negative autoregulation of ribosomal protein gene expression.

Class C, comprising genes that are differentially regulated only in the *rel*-deficient mutant include a number of genes encoding transcriptional regulators. This can be interpreted as an expression of the fact that the cellular metabolism in the Δ*rel *mutant is much more unbalanced. It seems to be clear that the existence of the latter two classes of genes implies not a certain mechanism for stringent control only active when the Rel protein is inactive or absent. In contrast, it seems to be the expression of an imbalanced metabolism trying to antagonize the effects of an artificial amino acid deficiency without its master regulator (p)ppGpp.

In the longer run this might lead to the complex pattern of differential gene expression observed when comparing the transcriptomes of the *rel *mutant directly with that of its *rel*-proficient parent strain.

## Materials and methods

### Bacterial strains and growth conditions

In this work, *C. glutamicum *RES167, a restriction and modification-deficient derivative of the wild-type strain ATCC 13032 [51] and *C. glutamicum *RES167Δ*rel *[6] were used. The strains were cultivated at 30°C in Luria-Bertani medium [52] supplemented with 2 gl^-1 ^glucose (LBG). For transcriptome analysis the cultures were grown to an O.D._600 _of 0.2 and then harvested. In the induction experiments, the cultures were divided into two aliquots, one of which was treated with DL-serine hydroxamate (final concentration 50 mM) to induce the stringent response.

### Total RNA isolation from *C. glutamicum*

Approximately 1 × 10^9 ^cells were harvested from *C. glutamicum *cultures and immediately frozen in a dry ice/ethanol bath. The cells were thawed and centrifuged at 6.000 g for 60 seconds. The supernatant was decanted and the pellet was resuspended in 800 μl RLT-buffer (RNeasy Mini Kit, Qiagen) and disrupted by means of a Rybolyser Instrument (Hybaid, Heidelberg, Germany). The following clean-up of RNA along with DNase I digestion was performed as described [9].

### cDNA synthesis, DNA microarray experiments and data analysis

The labeled cDNA was synthezised according to [9], as were the DNA microarray experiments and data analysis. The ImaGene 5.0 Software (BioDiscovery, Los Angeles, CA) was used for spot finding, signal-background segmentation and intensity quantification. Normalization and *t*-test statistics were carried out with the EMMA microarray data analysis software [53]. According to the validation experiments [9] all genes with detected gene expression ratios greater than 2^0.6 ^or smaller than 2^-0.6 ^were regarded as differentially expressed with a confidence level of at least 95%. The additional Students *t*-test statistics were performed with a probability cutoff of p = 0.05.

### Real-time RT-PCR assays

One-step real-time RT-PCR reactions were performed by using the LightCycler instrument (Roche Diagnostics) and the QuantiTect SYBR Green RT-PCR Kit (Qiagen) according to the manufacturer's instructions. To verify the purity of the obtained PCR products, melting curve analyses were performed. Differences in gene expression were calculated by comparing the crossing points of the samples with the help of the LightCycler software (version 3; Roche Diagnostics).

## Authors' contributions

OBG carried out the experimental work and drafted the manuscript. JK conceived and co-ordinated the study and participated in writing. All authors read and approved the final manuscript.

## Supplementary Material

Additional File 1 **Pivot table of the differentially expressed genes of the *C. glutamicum rel*-proficient strain and its derived *rel*-deletion mutant in the presence and absence of serine hydroxamate**. Relevant expression data of all genes differentially expressed in the comparison of *C. glutamicum *RES167 and its derived *rel*-deletion strain in the presence and absence of serine hydroxamate. Empty fields represent non-significant expression ratios. Click here for file

## Figures and Tables

**Table 1:** Class A: Genes under Rel-dependent stringent control

**Locus tag**^*a*^	**ratio**	**gene name**	**Annotation**	**COG class**^*b*^
**Genes under Rel-dependent, negative stringent control**				

cg0104	0.63	*codA*	creatinine deaminase	FR
cg0113	0.66	*ureA*	urease (γ subunit)	E
cg0229	0.33	*gltB*	glutamine 2-oxoglutarate aminotransferase large subunit	E
cg0291	0.62		3,4-dioxygenase beta subunit	Q
cg0419	0.59		glycosyltransferase	M
cg0599	0.58	*rpsS*	30S ribosomal protein S19	J
cg0637	0.55	*betB*	betaine aldehyde dehydrogenase oxireductase	C
cg0990	0.65	*rpmG*	50S ribosomal protein L33	J
cg1062	0.43	*urtB*	ABC-type urea transport system, permease component	E
cg1064	0.65	*urtC*	ABC-type urea transport system, permease component	E
cg1341	0.62	*narI*	respiratory nitrate reductase 2 (γ chain)	C
cg1342	0.64	*narJ*	nitrate reductase (Δ chain)	C
cg1606	0.62	*pyrG*	CTP synthetase	F
cg1607	0.66		NTP pyrophosphohydrolase	LR
cg1783	0.29	*soxA'*	sarcosine oxidase, N-terminal fragment	E
cg1784	0.50	*ocd*	ornithine cyclodeaminase	E
cg1785	0.33	*amt*	high-affinity ammonia permease	P
cg2260	0.27	*glnK*	Nitrogen regulatory protein PII	E
cg2280	0.39	*gdh*	NADP-specific glutamate dehydrogenase	E
cg2429	0.52	*glnA*	glutamine synthetase I	E
cg2942	0.48		Bacterial regulatory proteins, AsnC family	K
cg3308	0.65	*rpsF*	30S ribosomal protein S6	J
cg3370	0.58		NADH-dependent flavin oxidoreductase	C

**Genes under Rel-dependent, positive stringent control**				

cg0414	1.66	*wzz*	cell surface polysaccharide biosynthesis protein	D
cg0552	1.60	*menD*	2-oxoglutarate decarboxylase	H
cg0834	1.58		Bacterial extracellular solute-binding protein	G
cg0924	3.24		ABC-type cobalamin/Fe3+-siderophores transport system	P
cg0926	1.87		ABC-type cobalamin/Fe3+-siderophores transport system	P
cg0928	1.64		ABC-type cobalamin/Fe3+-siderophores transport system	P
cg1873	1.69	*tesB2*	acyl-CoA thioesterase II	I
cg2102	1.79	*sigB*	RNA polymerase sigma factor	K
cg2114	1.59	*lexA*	LexA repressor transcriptional regulator	KT
cg2120	1.53	*ptsF*	PTS system, fructose/mannitol-specific enzyme II	G
cg2291	1.53	*pyk*	pyruvate kinase	G
cg2398	1.55	*plsC*	1-acyl-SN-glycerol-3-phosphate acetyltransferase	I
cg2403	2.61	*qcrB*	cytochrome B	C
cg2405	1.69	*qcrC*	cytochrome C1	C
cg2406	1.63	*ctaE*	cytochrome C, oxidase subunit 3	C
cg2459	1.53	*ptpA*	protein-tyrosine phosphatase	T
cg2645	1.82	*clpP1*	ATP-dependent Clp protease, proteolytic subunit ClpP1	OU
cg2963	2.09	*clpC*	ATP-dependent Clp protease ATPase subunit	O
cg3219	1.77	*ldh*	L-lactate dehydrogenase	C

*a *Locus tag according to [50]

*b *COG class according to [11]

**Table 2:** Class B: Genes under Rel-independent control

**Locus tag**^*a*^	**ratio (WT)**	**ratio (Δrel)**	**gene name**	**Annotation**	**COG class**^*b*^
**Genes under Rel-independent, negative control**					

cg0464	0.56	0.40	*ctpA*	copper-transporting ATPase	P
cg0581	0.58	0.45	*rpsL*	30S ribosomal protein S12	J
cg0582	0.41	0.43	*rpsG*	30S ribosomal protein S7	J
cg0593	0.53	0.31	*rpsJ*	30S ribosomal protein S10	J
cg0594	0.52	0.44	*rplC*	50S ribosomal protein L3	J
cg0597	0.44	0.26	*rplW*	50S ribosomal protein L23	J
cg0600	0.54	0.31	*rplV*	50S ribosomal protein L22	J
cg0602	0.58	0.28	*rplP*	50S ribosomal protein L16	J
cg0604	0.46	0.47	*rpsQ*	30S ribosomal protein S17	J
cg0608	0.49	0.26	*rplN*	50S ribosomal protein L14	J
cg0609	0.59	0.45	*rplX*	50S ribosomal protein L24	J
cg0610	0.49	0.34	*rplE*	50S ribosomal protein L5	J
cg0628	0.42	0.34	*rpsH*	30S ribosomal protein S8	J
cg0629	0.53	0.48	*rplF*	50S ribosomal protein L6	J
cg0632	0.57	0.19	*rpmD*	50S ribosomal protein L30	J
cg0634	0.43	0.19	*rplO*	50S ribosomal protein L15	J
cg0651	0.56	0.31	*infA*	translation initiation factor IF-1	J
cg0652	0.61	0.40	*rpsM*	30S ribosomal protein S13	J
cg0655	0.57	0.38	*rpoA*	DNA-directed RNA polymerase (α subunit)	K
cg0656	0.60	0.43	*rplQ*	50S ribosomal protein L17	J
cg0673	0.43	0.43	*rplM*	50S ribosomal protein L13	J
cg0756	0.54	0.48	*cstA*	carbon starvation protein A	T
cg0994	0.62	0.41	*rpmE*	50S ribosomal protein L31	J
cg1343	0.57	0.47	*narH*	respiratory nitrate reductase oxireductase	C
cg1344	0.60	0.41	*narG*	nitrate reductase 2 (α subunit)	C
cg1345	0.59	0.37	*narK*	nitrate/nitrite transporter	P
cg1565	0.66	0.62	*rplT*	50S ribosomal protein L20	J
cg1623	0.65	0.50		divalent heavy-metal cations transporter	P
cg2167	0.46	0.56	*rpsO*	30S ribosomal protein S15	J
cg2253	0.53	0.49	*rpsP*	30S ribosomal protein S16	J
cg2573	0.41	0.30	*rpsT*	30S ribosomal protein S20	J
cg2595	0.65	0.64	*rplU*	50S ribosomal protein L21	J
cg3140	0.13	0.35	*tagA1*	DNA-3-methyladenine glycosylase I	L
cg3141	0.22	0.46	*hmp*	NO-detoxification flavohemoprotein	C
cg3306	0.62	0.41	*rplI*	50S ribosomal protein L9	J
cg3307	0.44	0.42	*ssb*	single-stranded DNA-binding protein	L

**Genes under Rel-independent, positive control**					

cg0310	3.12	4.89	*katA*	catalase	P
cg0812	1.66	2.13	*dtsR1*	acetyl/propionyl-CoA carboxylase (β chain)	I
cg0898	3.33	2.75		pyridoxine biosynthesis enzyme	H
cg0899	3.29	3.30		glutamine amidotransferase (involved in pyridoxine biosynthesis)	H
cg1761	1.55	3.81	*nifS2*	cysteine desulfhydrase	E
cg1763	1.72	6.59	*sufD*	components of an uncharacterized iron-regulated ABC-type transporter	O
cg1764	1.79	2.43	*sufB*	component of an uncharacterized iron-regulated ABC-type transporter	O
cg2409	2.31	1.61	*ctaC*	cytochrome C oxidase chain II	C
cg2644	1.83	2.93	*clpP2*	ATP-dependent Clp protease, proteolytic subunit	OU
cg2732	1.68	2.41	*gntV*	gluconokinase	G
cg3114	1.55	2.90	*cysN*	sulfate adenyltransferase subunit 1	P
cg3327	3.03	2.89	*dps*	starvation-induced DNA protecting protein	P

*a L*ocus tag according to [50]

*b *COG class according to [11]
