# Supplementary material for: Global gene expression during stringent response in Corynebacterium glutamicum in presence and absence of the rel gene encoding (p)ppGpp synthase
Source: BMC Genomics. 2006 Sep 8;7:230. doi: 10.1186/1471-2164-7-230 (PMC1578569; doi:10.1186/1471-2164-7-230)
Supplement: Additional File 1 — Pivot table of the differentially expressed genes of the C. glutamicum rel-proficient strain and its derived rel-deletion mutant in the presence and absence of serine hydroxamate. Relevant expression data of all genes differentially expressed in the comparison of C. glutamicum RES167 and its derived rel-deletion strain in the presence and absence of serine hydroxamate. Empty fields represent non-significant expression ratios. [file 1471-2164-7-230-S1.pdf]

Locus tag[a]	ratio (Drel/RES)	ratio (RES_{t10}/RES_{t0})	ratio ($Drel_{t10}/Drel_{t0}$)	gene name	annotation	COG class[b]
cg0007	2,02			*gyrB*	DNA gyrase subunit B	L
cg0047	0,12				Conserved hypothetical protein	M
cg0049	0,50				Putative membrane protein, Rhomboid-family	R
cg0057		1,54		*pknB*	Serine/threonine protein kinase	RTKL
cg0064			0,44		Conserved hypothetical protein	T
cg0065			0,56		Hypothetical protein	
cg0077	3,93				Conserved hypothetical protein	
cg0078	5,31				Putative membrane protein	
cg0079	3,24				Putative secreted protein	M
cg0081	1,97				Putative tautomerase	R
cg0082	1,64				Chloride ion channel, ClC-family	P
cg0088	0,57			*citP*	Putative secondary Mg2+/H+:citrate transporter, CitMHS-family	C
cg0107	2,23				Putative secreted protein	NU
cg0113		0,63		*ureA*	Urease gamma subunit	E
cg0129	1,68			*putA*	Proline dehydrogenase/delta-1-pyrroline-5-carboxylate dehydrogenase	C
cg0138			1,73		ATP/GTP-binding protein	
cg0154	1,54				Putative hydrolase	R
cg0161			1,70		Putative membrane protein	
cg0175	1,89				Putative secreted protein	
cg0177	1,54				Hypothetical protein	
cg0194			1,54		Putative secreted protein	
cg0215	0,59	0,64	0,63	*cspA*	Cold-shock protein A	K
cg0216	0,63				Putative membrane protein	
cg0221	0,58				Putative transcriptional regulator, LacI-family	K
cg0229		0,37		*gltB*	Glutamate synthase (NADPH), large chain	E
cg0238	1,68		1,72		FAD/FMN-containing dehydrogenase	C
cg0242			3,21		Hypothetical protein	
cg0249		0,61			ABC-type putative polysaccharide transporter, permease subunit	GM
cg0281			0,64		tRNA-specific adenosine deaminase	FJ
cg0291	2,50	0,62			Putative dioxygenase	Q
cg0297		1,70			Conserved hypothetical protein, DUF149-family	S
cg0306	1,72			*lysC*	Aspartate kinase	E
cg0307	1,87			*asd*	Aspartate-semialdehyde dehydrogenase	E
cg0310	0,46	3,39	4,90	*katA*	Catalase	P
cg0337			0,59	*whiB4*	Putative transcriptional regulator, WhiB-family	
cg0352	1,61				Putative secreted protein	
cg0359	2,00				Putative membrane protein	
cg0378	1,54				Putative phage-associated protein	S
cg0387			1,83		Putative NAD/mycothiol-dependent formaldehyde dehydrogenase	C
cg0394			0,59		Putative glycosyl transferase	M
cg0400			1,63	*adhC*	Putative alcohol dehydrogenase (NADP(+))	R
cg0414		1,68		*wzz*	Cell surface polysaccharide biosynthesis/chain length determinant	D
cg0416			1,67		Putative secreted protein, carrying a eukaryotic domain	
cg0419	0,59	0,63			Putative glycosyltransferase	M
cg0431	0,56				Putative membrane protein, involved in polysaccharide	I

					acetylation	
cg0435	0,48		0,47	*udgA1*	UDP-glucose 6-dehydrogenase	M
cg0438			0,66		Putative glycosyltransferase	M
cg0441		0,63		*lpd*	Dihydrolipoamide dehydrogenase	C
cg0442	0,58		0,66	*galU2*	Putative UTP--glucose-1-phosphate uridylyltransferase	M
cg0448			0,56		Conserved putative membrane protein	
cg0450			1,74		Conserved hypothetical protein	
cg0453	1,53				Putative membrane protein	
cg0464	0,63	0,62	0,40	*ctpA*	Putative Cu2+ transporting P-type ATPase	P
cg0467	2,12				ABC-type putative hemin transporter, substrate-binding lipoprotein	P
cg0475	1,91				Conserved hypothetical protein	
cg0489	1,61				Putative membrane protein	
cg0490			1,62	*proC*	Pyrroline-5-carboxylate reductase	E
cg0513	1,98				Putative membrane protein	
cg0527	1,75				Putative transcriptional regulator, ArsR-family	K
cg0528	2,42				Putative secreted protein	
cg0538	2,63				Conserved hypothetical protein	
cg0552		1,78		*menD*	2-Oxoglutarate decarboxylase	H
cg0563			0,31	*rplK*	50S ribosomal protein L11	J
cg0564	1,76		0,43	*rplA*	50S ribosomal protein L1	J
cg0569	1,93				Putative Cd2+ transporting P-type ATPase	P
cg0576	1,57		0,65	*rpoB*	DNA-directed RNA polymerase, beta chain	K
cg0581		0,56	0,45	*rpsL*	30S ribosomal protein S12	J
cg0582		0,31	0,43	*rpsG*	30S ribosomal protein S7	J
cg0587			2,07	*tuf*	Elongation factor Tu	J
cg0593		0,40	0,31	*rpsJ*	30S ribosomal protein S10	J
cg0594		0,38	0,44	*rplC*	50S ribosomal protein L3	J
cg0597	2,41	0,35	0,29	*rplW*	50S ribosomal protein L23	J
cg0597	1,61	0,36	0,26	*rplW*	50S ribosomal protein L23	J
cg0599	1,76	0,54		*rpsS*	30S ribosomal protein S19	J
cg0600		0,46	0,31	*rplV*	50S ribosomal protein L22	J
cg0601	2,95		0,30	*rpsC*	30S ribosomal protein S3	J
cg0602	1,86	0,49	0,28	*rplP*	50S ribosomal protein L16	J
cg0603	1,65	0,56	0,26	*rpmC*	50S ribosomal protein L29	J
cg0604	2,11	0,43	0,47	*rpsQ*	30S ribosomal protein S17	J
cg0608		0,41	0,26	*rplN*	50S ribosomal protein L14	J
cg0609		0,48	0,45	*rplX*	50S ribosomal protein L24	J
cg0610		0,43	0,34	*rplE*	50S ribosomal protein L5	J
cg0620			0,57		Putative secreted protein	
cg0628		0,46	0,34	*rpsH*	30S ribosomal protein S8	J
cg0629		0,42	0,48	*rplF*	50S ribosomal protein L6	J
cg0630		0,61	0,29	*rplR*	50S ribosomal protein L18	J
cg0631		0,62	0,18	*rpsE*	30S ribosomal protein S5	J
cg0632		0,51	0,19	*rpmD*	50S ribosomal protein L30	J
cg0634		0,43	0,19	*rplO*	50S ribosomal protein L15	J
cg0637		0,60		*betB*	Putative betaine aldehyde dehydrogenase (BADH)	C
cg0648			1,53	*adk*	Adenylate kinase	F
cg0650			1,67		Putative secreted protein	S
cg0651		0,46	0,31	*infA*	Translation initiation factor IF-1	J
cg0652		0,52	0,40	*rpsM*	30S ribosomal protein S13	J
cg0653	1,65	0,60	0,51	*rpsK*	30S ribosomal protein S11	J
cg0654		0,56	0,33	*rpsD*	30S ribosomal protein S4	J
cg0655	1,65	0,57	0,38	*rpoA*	DNA-directed RNA polymerase, alpha subunit	K

cg0656	1,65	0,54	0,43	*rplQ*	50S ribosomal protein L17	J
cg0673	1,55	0,51	0,43	*rplM*	50S ribosomal protein L13	J
cg0674	2,12		0,65	*rpsI*	30S ribosomal protein S9	J
cg0675			0,58	*mrsA*	Putative phosphoglucosamine mutase/phosphomannomutase	G
cg0690	1,85			*groES*	10kDa chaperonin	O
cg0691	2,16			*groEL'*	60kDa chaperonin, putative pseudogene (N-terminal fragment)	O
cg0693	1,58			*'groEL*	60kDa chaperonin, putative pseudogene (C-terminal fragment)	O
cg0696	0,27			*sigD*	RNA polymerase sigma factor, ECF-family	K
cg0699	1,64			*guaB2*	IMP dehydrogenase	F
cg0700			0,64	*guaB3*	IMP dehydrogenase/GMP reductase	F
cg0701			0,62		Putative secondary drug/metabolite transporter, drug/metabolite transporter (DMT) superfamily	R
cg0703	1,76			*guaA*	Putative GMP synthase	F
cg0706	3,95				Conserved putative membrane protein	KT
cg0712	1,66				Putative secreted protein	
cg0717			1,77	*crtEb*	Lycopene elongase	H
cg0720			1,64	*crtI2*	Phytoene dehydrogenase (desaturase)	Q
cg0725	0,64		0,64		Putative transcriptional regulator, MarR-family	K
cg0752	1,93				Putative secreted or membrane protein	S
cg0753	1,99				Putative secreted protein	
cg0754	1,84	0,59		*metX*	Homoserine O-acetyltransferase	E
cg0755	1,62			*metY*	O-Acetylhomoserine sulfhydrylase	E
cg0756	0,60	0,47	0,48	*cstA*	Putative carbon starvation protein A	T
cg0766	1,64		1,71	*icd*	Isocitrate dehydrogenase	C
cg0773			1,55		Putative exodeoxyribonuclease	L
cg0774	0,64				Putative membrane protein	S
cg0775	0,56				Hypothetical protein	
cg0778	0,55				ABC-type putative iron-siderophore transporter, permease subunit	P
cg0781	3,71				Putative membrane protein	
cg0791	0,50			*pyc*	Pyruvate carboxylase	C
cg0793	0,59				Putative secreted protein	S
cg0811	0,50			*dtsR2*	Acetyl/propionyl-CoA carboxylase, beta chain	I
cg0812	0,28	1,52	2,13	*dtsR1*	Acetyl/propionyl-CoA carboxylase, beta chain	I
cg0834		1,66			ABC-type putative sugar transporter, substrate-binding lipoprotein	G
cg0841		0,64			Conserved hypothetical protein	S
cg0842	0,58				Putative DNA helicase	L
cg0849			0,61	*rmlA2*	Mannose-1-phosphate guanylyltransferase (GDP)	MJ
cg0850			0,61	*whiB2*	Putative transcriptional regulator, WhiB-family	
cg0853			0,63		Conserved hypothetical protein	
cg0858			1,58		Putative secreted protein	S
cg0865		0,61			Putative secreted lipoprotein	
cg0866	0,57				Conserved hypothetical protein	R
cg0867	2,38		2,16		Putative ribosome-associated protein Y (PSrp-1)	J
cg0878	0,31			*whiB1*	Putative transcriptional regulator, WhiB-family	
cg0882	0,57	0,64			Conserved hypothetical protein	
cg0883			1,57		Conserved putative secreted	

					protein	
cg0885		0,62			Putative helicase, UvrD/Rep-family	L
cg0892		1,59			Conserved hypothetical protein	S
cg0893			0,63		Putative secreted protein, containing a PDZ-domain	T
cg0898	0,35	3,33	2,75		Pyridoxine biosynthesis enzyme	H
cg0899	0,39	3,55	3,31		Putative glutamine amidotransferase, involved in pyridoxine biosynthesis	H
cg0904			1,80		Hypothetical protein	
cg0910	0,64		1,59	*himP*	Histidinol-phosphatase, ImpA-family	G
cg0913			0,64	*prfB*	Peptide chain release factor 2 (RF-2)	J
cg0922	0,65				ABC-type putative iron-siderophore transporter, substrate-binding lipoprotein	P
cg0924		4,34			ABC-type putative iron-siderophore transporter, substrate-binding lipoprotein	P
cg0926		1,84			ABC-type putative iron-siderophore transporter, permease subunit	P
cg0928		1,85			ABC-type putative iron-siderophore transporter, ATPase subunit	P
cg0936	0,15			*rpf1*	RPF-protein precursor	
cg0938			0,64		Cold shock protein	K
cg0939			0,65		Putative secreted protein	
cg0952	1,83	2,29			Putative integral membrane protein	S
cg0953	1,99	2,66			Putative Na+/solute symporter, solute:sodium symporter (SSS) family	R
cg0957	0,57			*fas-IB*	Fatty acid synthase	I
cg0980	0,29				Putative secreted protein, related to metalloendopeptidases	M
cg0984	1,59			*purH*	Phosphoribosylaminoimidazolecar boxamide formyltransferase	F
cg0988		0,60	0,34	*rpsR*	30S ribosomal protein S18	J
cg0989		0,60	0,39	*rpsN*	30S ribosomal protein S14	J
cg0990			0,41	*rpmG*	50S ribosomal protein L33	J
cg0991			0,42	*rpmB*	50S ribosomal protein L28	J
cg0994		0,59	0,41	*rpmE*	Putative 50S ribosomal protein L31	J
cg0998	6,22				Trypsin-like serine protease	O
cg0999	2,20				Putative molybdopterin biosynthesis protein	H
cg1010			0,60		Putative membrane protein	S
cg1013		1,55			Hypothetical protein	
cg1016			0,60	*betP*	Putative secondary glycine betaine/choline transporter, betaine/carnitine/choline transporter (BCCT) family	M
cg1037	0,40			*rpf2*	RPF2 precursor, secreted protein	S
cg1038	0,65		0,64	*ksgA*	Putative dimethyladenosine transferase	J
cg1039	0,61		0,62		Putative isopentenyl monophosphate kinase	I
cg1049		0,65			Putative enoyl-CoA hydratase/isomerase	I
cg1055	1,78			*menG*	S-Adenosylmethionine:2-demethylmenaquinone methyltransferase	H
cg1057	0,66				Hypothetical protein	S
cg1061	0,65			*urtA*	ABC-type putative branched-chain amino acid transporter, substrate-binding lipoprotein	E
cg1062		0,46		*urtB*	ABC-type putative branched-chain amino acid transporter, permease subunit	E

cg1068	1,92				Putative oxidoreductase	R
cg1071	1,79			*pth1*	Putative peptidyl-tRNA hydrolase	J
cg1072			0,56	*rplY*	Ribosomal protein L25 (general stress protein Ctc)	J
cg1075	2,37			*prsA*	Ribose-phosphate diphosphokinase	FE
cg1080	1,69				Putative multicopper oxidase	Q
cg1091	0,41				Hypothetical protein	
cg1095	0,48		0,56		Hypothetical protein	
cg1097	0,58		0,65		Hypothetical protein	
cg1099		0,65		*mfd*	Transcription-repair coupling factor (TRCF)	LK
cg1104	1,83				Putative membrane protein, predicted esterase	R
cg1109			0,53		Hypothetical protein	
cg1110	2,10		1,56		Conserved hypothetical protein	M
cg1113			1,57		Conserved hypothetical protein	S
cg1115			0,61	*ppx2*	Putative exopolyphosphatase	FP
cg1121	1,62				Permease, MFS-type	S
cg1122	1,55				Putative secreted protein	
cg1125	1,69				Conserved hypothetical protein	
cg1125	1,68				Conserved hypothetical protein	
cg1129	0,55			*aroF*	Putative phospho-2-dehydro-3-deoxyheptonate aldolase	E
cg1130	1,76			*uppS1*	Putative undecaprenyl pyrophosphate synthetase	I
cg1131	1,69				Conserved hypothetical protein	
cg1136			1,67		Conserved hypothetical protein	
cg1138	0,49				Putative acetyltransferase, GNAT-family	M
cg1139	0,60		0,41		Allophanate hydrolase subunit 2	E
cg1140			0,50		Allophanate hydrolase subunit 1	E
cg1141			0,46		Conserved hypothetical protein, UPF0271-family	R
cg1142	0,37		0,49		Putative Mn2+ transporter, metal ion (Mn2+-iron) transporter (Nramp) family	P
cg1147	1,62			*ssuI*	NAD(P)H-dependent FMN reductase	R
cg1150	0,40				Putative NADPH-dependent FMN reductase	R
cg1157			1,89	*glpX*	Fructose-1,6-bisphosphatase II	G
cg1158	0,55		0,54		Putative secreted protein	
cg1171	1,78				Putative GTPase	J
cg1181			1,89		Glycosyltransferase, probably involved in cell wall biogenesis	M
cg1201	0,65				Hypothetical protein	
cg1203			1,94		Putative magnesium chelatase, ChlI subunit	H
cg1228	1,53				ABC-type putative cobalt transporter, ATPase subunit	P
cg1236			2,27	*tpx*	Thiol peroxidase	O
cg1245	0,59				Putative membrane protein	S
cg1246	0,64				Conserved hypothetical protein	S
cg1247	0,55				Putative secreted protein	
cg1255			1,64		Conserved hypothetical protein, putative HNH endonuclease	
cg1263	0,65				Glycosyltransferase, involved in cell wall biogenesis	M
cg1265			0,60		Conserved hypothetical protein	
cg1271	4,32			*sigE*	RNA polymerase sigma factor, ECF-family	K
cg1277	2,68				Conserved putative membrane protein	
cg1278	2,15				Conserved putative secreted protein	
cg1279	2,69				Putative secreted protein	

cg1280	1,54		1,74	*odhA*	2-Oxoglutarate dehydrogenase, E1 component	C
cg1283			1,68	*aroE2*	Putative shikimate/quinate 5-dehydrogenase	E
cg1284			1,54	*lipT*	Putative carboxylesterase, type B	I
cg1290		1,56		*metE*	5-Methyltetrahydropteroyltriglutamate--homocysteine methyltransferase	E
cg1291		1,88	1,86		Putative membrane protein	
cg1293		1,56			Putative secreted protein	
cg1300			1,98	*cydB*	Cytochrome d ubiquinol oxidase subunit II	C
cg1301			1,70	*cydA*	Cytochrome d ubiquinol oxidase subunit I	C
cg1304	0,53				Putative secreted protein	
cg1307	0,43		1,97		DNA/RNA helicase, superfamily II	LKJ
cg1307	0,32				DNA/RNA helicase, superfamily II	LKJ
cg1314	2,21			*putP*	Putative Na+/proline symporter, solute:sodium symporter (SSS) family	ER
cg1324	2,08				Putative transcriptional regulator, MarR-family	K
cg1325	2,52				Conserved hypothetical protein	KT
cg1327	2,08				Putative transcriptional regulator, Crp-family	T
cg1330	0,66				Conserved hypothetical protein	S
cg1337	1,65			*hom*	Homoserine dehydrogenase	E
cg1338	1,53			*thrB*	Homoserine kinase	E
cg1341		0,56		*narI*	Respiratory nitrate reductase 2, gamma chain	C
cg1342		0,59		*narJ*	Respiratory nitrate reductase 2, delta chain	C
cg1343		0,65	0,47	*narH*	Respiratory nitrate reductase 2, beta chain	C
cg1344	0,58	0,54	0,41	*narG*	Respiratory nitrate reductase 2, alpha chain	C
cg1345		0,57	0,37	*narK*	Putative nitrate/nitrite permease, MFS-type	P
cg1346			0,58	*mog*	Putative molybdopterin biosynthesis protein Mog	H
cg1364			1,57	*atpF*	ATP synthase F0, B chain	C
cg1365			1,55	*atpH*	ATP synthase F1, delta subunit	C
cg1366			2,29	*atpA*	ATP synthase F1, alpha chain	C
cg1367	1,53		0,65	*atpG*	ATP synthase F1, gamma chain	C
cg1368	2,13			*atpD*	ATP synthase F1, beta chain	C
cg1370	0,62				Conserved hypothetical protein	
cg1373	2,36				Putative glyoxalase	E
cg1387			1,99	*fixB*	Putative electron transfer flavoprotein, alpha subunit	C
cg1391	1,67				Conserved hypothetical protein, related to capsule biosynthesis enzymes	R
cg1397			0,53	*trmU*	tRNA (5-methylaminomethyl-2-thiouridylate)-methyltransferase	J
cg1409			1,52	*pfkA*	6-Phosphofructokinase	G
cg1417	1,62				Putative acetyltransferase	J
cg1419		0,66			Putative secondary Na+/bile acid symporter, bile acid:Na+ symporter (BASS) family	R
cg1421	2,18				Conserved hypothetical protein, putative dinucleotide-binding enzyme	R
cg1421		0,64			Conserved hypothetical protein, putative dinucleotide-binding enzyme	R
cg1429			1,52		Putative membrane protein	S
cg1454	0,58				ABC-type putative aliphatic sulfonates transporter, substrate-binding lipoprotein	P

cg1458	1,69				Putative hydrolase, FAA-family	Q
cg1459	1,55				Putative SAM-dependent methyltransferase	QR
cg1478	1,80				Hypothetical protein	
cg1479	0,61			*glgP1*	Putative glycogen phosphorylase	G
cg1483	1,53				Putative membrane protein	R
cg1484			1,59		Putative secreted protein	
cg1511	0,58				Hypothetical protein	
cg1514	1,77		1,73		Putative secreted protein	M
cg1516			0,65		Hypothetical protein	
cg1517			0,55		Putative secreted protein	
cg1537			0,46	*ptsG*	Phosphotransferase system (PTS), glucose-specific enzyme IIBCA component	G
cg1542	0,64				Putative membrane protein	
cg1547	2,27			*ccpA1*	Putative transcriptional regulator, LacI-family	K
cg1564	0,65			*rpmI*	50S ribosomal protein L35	J
cg1565		0,63	0,63	*rplT*	50S ribosomal protein L20	J
cg1567			0,62		Hypothetical protein	
cg1580	1,74			*argC*	N-acetyl-gamma-glutamyl-phosphate reductase	E
cg1586	1,57			*argG*	Argininosuccinate synthase	E
cg1606	1,55	0,47		*pyrG*	CTP synthetase	F
cg1607	1,52	0,58			Putative NTP pyrophosphohydrolase	LR
cg1613	1,85			*sseA2*	Rhodanese-related sulfurtransferase	P
cg1615	0,63				16S rRNA uridine-516 pseudouridylate synthase or related pseudouridylate synthase	J
cg1616	0,66		0,53	*cmk*	Cytidylate kinase	F
cg1617	0,59				GTPase of unknown function	R
cg1623			0,50		Putative divalent heavy-metal cation transporter	P
cg1624	1,75	1,56			Putative secondary Na+/H+ antiporter, monovalent cation:proton antiporter-1 (CPA1) family	P
cg1626		1,69	2,39		Conserved hypothetical protein	S
cg1628			1,88		Putative hydrolase, alpha/beta superfamily	R
cg1630			1,54		Putative signal transduction protein, FHA -domain	T
cg1631	1,66		1,70		Putative transcriptional regulator, MerR-family	K
cg1635	2,00				Putative membrane protein	
cg1643	0,50		6,10	*gnd*	Phosphogluconate dehydrogenase (decarboxylating)	G
cg1646			1,62		ABC-type multidrug transport system, ATPase subunit	V
cg1649	1,59			*pctD*	ABC-type phosphate/phosphonate transporter, permease subunit (TC 3.A.1.9.1)	P
cg1650	2,05			*pctC*	ABC-type phosphate/phosphonate transporter, permease subunit (TC 3.A.1.9.1)	P
cg1651	3,21			*pctB*	ABC-type phosphate/phosphonate transporter, ATPase subunit (TC 3.A.1.9.1)	P
cg1652	5,80			*pctA*	ABC-type alkylphosphonate transporter, substrate-binding lipoprotein (TC 3.A.1.9.1)	P
cg1653	1,98			*pgp1*	Putative phosphoglycolate phosphatase	R
cg1662	2,20				Putative secreted protein	
cg1671	0,46		0,42		Putative membrane-associated GTPase	
cg1672			0,61	*ppmC*	Polyprenol-phosphate-mannose synthase domain 1	M

cg1684	1,71			*tatC*	Putative twin arginine targeting (Tat) Preprotein translocase subunit	U
cg1685	1,76			*tatX*	Putative twin arginine targeting (Tat) Preprotein translocase subunit	U
cg1693			1,75	*pepC*	Aspartyl aminopeptidase	E
cg1694	0,64			*recB*	Exonuclease, RecB-family	L
cg1707	0,53				Putative arsenate reductase (arsenical pump modifier)	T
cg1709		0,66		*mshC*	Putative 1-D-myo-inosityl-2-amino-2-deoxy-alpha-D-glucopyranoside--L-cysteine ligase	J
cg1711		0,57			Putative oxidoreductase	C
cg1717	0,53				Putative membrane protein	
cg1718			2,27		Phospholipid-binding protein	R
cg1730			1,92		Putative secreted protease subunit, stomatin/prohibitin-like	O
cg1731	0,66				Membrane protein, implicated in regulation of membrane protease activity	OU
cg1737			2,03	*acn*	Aconitate hydratase	C
cg1739	1,82				Conserved hypothetical protein, containing a glutamine amidotransferase domain	F
cg1743	0,63				Conserved hypothetical protein	S
cg1759			2,82		Putative metal-sulfur cluster biosynthetic enzyme	R
cg1760	0,60		2,36		Protein involved in Fe-S cluster formation, NifU-family	C
cg1761	0,56	1,62	3,81	*nifS2*	Cysteine desulfhydrase, AT class IV/selenocysteine lyase	E
cg1762	0,61		3,70	*sufC*	FeS assembly ATPase, SufC-family	O
cg1763	0,61	1,53	6,60	*sufD*	FeS assembly membrane protein, SufD-family	O
cg1764	0,64	1,76	2,43	*sufB*	FeS assembly membrane protein, SufB-family	O
cg1765	0,50	1,61	1,72		Putative regulator protein	K
cg1773			0,65	*ctaB*	Polyprenyltransferase, cytochrome oxidase assembly factor	H
cg1776	1,56			*tal*	Transaldolase	G
cg1783		0,30		*soxA'*	Putative oxidase, pseudogene (N-terminal fragment)	E
cg1784		0,51		*ocd*	Putative ornithine cyclodeaminase	E
cg1785	1,85	0,28		*amt*	Putative secondary ammonium transporter, Amt-family	P
cg1790	0,61			*pgk*	Phosphoglycerate kinase	G
cg1792	0,54		0,60		Putative transcriptional regulator, WhiB-family	S
cg1801	1,88		0,59	*rpe*	Ribulose-5-phosphate-3-epimerase	G
cg1803	1,55			*fmt*	Methionyl-tRNA formyltransferase	J
cg1809	0,63				DNA-directed RNA polymerase subunit K/omega	K
cg1812	2,35		0,47	*pyrF*	Orotidine-5'-phosphate decarboxylase	F
cg1813	2,61			*carB*	Carbamoyl-phosphate synthase, large chain	EF
cg1814	1,60		0,60	*carA*	Carbamoyl-phosphate synthase, small chain	EF
cg1815	1,65			*pyrC*	Dihydroorotase	F
cg1817	0,58			*pyrR*	Conserved hypothetical protein	F
cg1833			1,61		ABC-type putative iron-siderophore transporter, ATPase subunit	P
cg1834	0,66				ABC-type putative iron-siderophore transporter, permease subunit	PH
cg1838			0,62	*alaS*	Alanyl-tRNA synthetase	J
cg1855	1,66			*hisS*	Histidyl-tRNA synthetase	J

cg1860	0,50		1,82		Putative membrane protein	
cg1873		1,66		*tesB2*	Acyl-CoA thioesterase II	I
cg1874	0,51				Putative membrane protein	
cg1875	1,61				Putative membrane protein	
cg1879	1,93				Putative HIT family hydrolase	FGR
cg1883		1,54			Putative secreted protein	S
cg1884	0,42				Putative membrane protein	R
cg1890	1,74	0,44	0,44		Hypothetical protein	
cg1891		0,49	0,28		Hypothetical protein	
cg1909	0,52				Hypothetical protein	
cg1911			0,66		Putative secreted protein	
cg1915	1,68				Hypothetical protein	
cg1921		0,64			Hypothetical protein	
cg1962			1,72		Putative membrane protein	
cg1966	0,64				Hypothetical protein	
cg1980	0,60				Hypothetical protein, MoxR-like ATPase	R
cg2015		0,65			Hypothetical protein	
cg2037	0,61				Conserved hypothetical protein	
cg2047	0,65				Putative secreted protein	
cg2052	0,55				Putative secreted protein	
cg2056	0,65				Putative membrane protein	
cg2057			1,70		Putative secreted protein	
cg2061	0,51		1,72	*psp3*	Putative secreted protein	
cg2066	0,65				Hypothetical protein, low-complexity protein	S
cg2079		0,64	0,57		Conserved hypothetical protein	S
cg2080			0,62		Conserved hypothetical protein	
cg2099	0,60				Putative membrane protein	
cg2102		1,80		*sigB*	RNA polymerase sigma factor rpoD (Sigma-A).	K
cg2103			1,61	*dtxR*	Putative transcriptional regulator, DtxR-family	K
cg2112	1,65				Putative transcriptional regulator, YbaD-family	K
cg2113	1,72				Hypothetical protein	
cg2114	1,53			*lexA*	Putative transcriptional regulator, LexA-family	KT
cg2115	1,64				Putative transcriptional regulator, DeoR-family	KG
cg2117			0,41	*ptsI*	Phosphotransferase system (PTS), Enzyme I	G
cg2118	0,48				Transcriptional regulator protein, DeoR-family	KG
cg2120	0,59			*ptsF*	Phosphotransferase system (PTS), fructose-specific enzyme IIABC component	G
cg2121			1,55	*ptsH*	Phosphotransferase system (PTS), phosphocarrier protein HPr	G
cg2124			0,53		Hypothetical protein	KT
cg2127	4,65				Hypothetical protein	
cg2136	0,61			*gluA*	ABC-type glutamate transporter, ATPase subunit (TC 3.A.1.3.9)	E
cg2141	1,58			*recA*	Recombinase A	L
cg2151			1,60		Conserved hypothetical protein, Similar to phage shock protein A	KT
cg2151			0,66		Conserved hypothetical protein, Similar to phage shock protein A	KT
cg2152			0,50		Putative transcriptional regulator, HTH_3-family	
cg2153	0,54		0,49		Conserved hypothetical protein, CinA-like protein	R
cg2160	0,57				Putative hydrolase of metallo-beta-lactamase superfamily	R
cg2163	1,84			*dapB*	Dihydrodipicolinate reductase	E
cg2165	2,25				Putative secreted protein	

cg2167		0,40	0,56	*rpsO*	30S ribosomal protein S15.	J
cg2170	0,63			*truB*	Pseudouridylate synthase	J
cg2176	0,66		0,47	*infB*	Translation initiation factor 2 (GTPase)	J
cg2177			0,57		Predicted nucleic-acid-binding protein implicated in transcription termination	K
cg2178			0,64	*nusA*	Putative transcriptional termination/antitermination factor	K
cg2181	2,91	0,56			ABC-type putative dipeptide/oligopeptide transporter, substrate-binding lipoprotein	E
cg2182	1,91				ABC-type putative dipeptide/oligopeptide transporter, permease subunit	EP
cg2183	1,75				ABC-type putative dipeptide/oligopeptide transporter, permease subunit	EP
cg2185	1,53			*proS*	Prolyl-tRNA synthetase	J
cg2195			1,57		Putative secreted or membrane protein	
cg2196		1,57			Putative secreted or membrane protein	
cg2214			1,53		Putative Fe-S-cluster redox enzyme	R
cg2215			1,52		Putative membrane protein	
cg2222			0,52	*rpsB*	30S ribosomal protein S2	J
cg2235			0,29	*rplS*	50S ribosomal protein L19	J
cg2253		0,65	0,49	*rpsP*	30S ribosomal protein S16	J
cg2254	0,58		0,60		Putative ankyrin repeat containing protein	R
cg2260		0,28		*glnK*	Nitrogen regulatory protein PII	E
cg2263			1,56		Hypothetical protein	
cg2275	1,80				Putative F0/F1-type ATP synthase b subunit	D
cg2277	1,74				ABC-type multidrug/protein/lipid transporter, permease subunit and ATPase subunit	V
cg2280		0,36		*gdh*	Glutamate dehydrogenase (NADP(+))	E
cg2289			2,29	*glgP2*	Phosphorylase	G
cg2291			1,69	*pyk*	Pyruvate kinase	G
cg2307			0,59		Putative membrane protein	
cg2308	1,93				Putative secreted protein	
cg2318	0,65				ABC-type putative iron(III) dicitrate transporter, substrate-binding lipoprotein	P
cg2323			1,73	*treY*	(1->4)-Alpha-D-glucan 1-alpha-D-glucosylmutase	G
cg2334			0,58	*ilvA*	Threonine ammonia-lyase	E
cg2343	0,60				Putative decarboxylase	S
cg2361	0,60				Cell division initiation protein - Antigen 84 homolog	D
cg2376			1,63		Putative secreted protein	
cg2377			1,77	*mraW*	S-adenosylmethionine-dependent methyltransferase involved in cell envelope biogenesis	M
cg2378	0,52		0,45	*mraZ*	Putative MarZ protein	S
cg2380			0,47		Putative membrane protein	
cg2401	0,63				Secreted protein NLP/P60 family, putative peptidoglycan lytic protein	M
cg2402	0,18				Secreted protein NLP/P60 family	M
cg2403		2,57		*qcrB*	Cytochrome b	C
cg2405			2,23	*qcrC*	Cytochrome c1	C
cg2406		1,78		*ctaE*	Cytochrome c oxidase subunit 3	C
cg2409		2,16	1,61	*ctaC*	Cytochrome c oxidase subunit II	C
cg2411		1,55			Conserved hypothetical protein, HesB/YadR/YfhF family	S
cg2417			2,21		Putative short-chain type	R

					oxidoreductase	
cg2418	1,61			*ilvE*	Branched-chain amino acid aminotransferase, AT class III	EH
cg2428	0,55				Conserved putative membrane protein	
cg2429		0,38		*glnA*	Glutamate--ammonia ligase	E
cg2430	1,65				Hypothetical protein	
cg2434			1,85		Putative monooxygenase, luciferase	C
cg2437			1,57	*thrC*	Threonine synthase	E
cg2440			1,60		Putative sugar/metabolite permease, MFS-type	GEPR
cg2443	2,25				Permease of the major facilitator superfamily	
cg2444	4,40	1,57	1,79		Hypothetical protein	
cg2445			1,80	*hmuO*	Heme oxygenase (decyclizing)	P
cg2459		1,63		*ptpA*	Protein-tyrosine-phosphatase	T
cg2462	0,63				Putative transcriptional regulator, TetR-family	
cg2466	1,54			*aceE*	Pyruvate dehydrogenase (acetyl-transferring)	C
cg2467			0,62		ABC-type transporter, ATPase subunit	R
cg2468			0,61		ABC-type transporter, permease subunit	R
cg2470	1,61				ABC-type transporter, substrate-binding lipoprotein	R
cg2474			1,79	*nagD*	Putative phosphatase involved in N-acetylglucosamine metabolism	G
cg2475			1,56		ABC-type transporter, ATPase subunit with duplicated ATPase domain	S
cg2477	0,47				Conserved hypothetical protein	
cg2499			1,63	*glyS*	Glycine--tRNA ligase	J
cg2500			1,76		Putative transcriptional regulator, ArsR-family	K
cg2502			1,64	*fur*	Putative transcriptional regulator, FUR-family	P
cg2513			1,56	*phoH2*	Phosphate starvation-inducible protein, PhoH-like	T
cg2518	0,55				Putative secreted protein	
cg2531		0,63		*idi*	Isopentenyl-diphosphate delta-isomerase	I
cg2533		0,65			Conserved hypothetical protein	
cg2535	1,87				Putative secreted protein	
cg2536			1,78	*aecD*	Beta C-S lyase, AT class I	E
cg2537			1,76	*brnQ*	Putative secondary branched-chain amino acid exporter, branched chain amino acid:cation symporter (LIVCS) family	E
cg2549	0,59				ABC-type putative dipeptide/oligopeptide transporter, substrate-binding lipoprotein	E
cg2564	0,44				Hypothetical protein	
cg2567	0,63		1,52		Hypothetical protein	
cg2573		0,47	0,30	*rpsT*	30S ribosomal protein S20	J
cg2594		0,65		*rpmA*	50S ribosomal protein L27	J
cg2595		0,58	0,64	*rplU*	50S ribosomal protein L21	J
cg2610		0,62			ABC-type putative dipeptide/oligopeptide transporter, substrate-binding lipoprotein	E
cg2613	3,06		2,01	*mdh*	Malate dehydrogenase	C
cg2614			1,75		Putative transcriptional regulator, TetR-family	
cg2630	0,62			*pcaG*	Protocatechuate 3,4-dioxygenase, alpha subunit	Q
cg2644	2,14	1,83	2,93	*clpP2*	Endopeptidase Clp, proteolytic subunit	OU
cg2645	2,10	2,02		*clpP1*	Endopeptidase Clp, proteolytic subunit	OU

cg2651	0,60				Conserved hypothetical protein, putative pseudogen	
cg2657	2,68				Putative membrane protein, putative pseudogen	
cg2662			1,91	*pepN*	Membrane alanyl aminopeptidase	E
cg2670		0,65		*crtI'*	Putative phytoene dehydrogenase, putative pseudogen (N-terminal fragment)	Q
cg2674	0,50		2,93		Alkylhydroperoxidase, AhpD-family core domain	S
cg2675	0,37		2,47		ABC-type putative dipeptide/oligopeptide transporter, ATPase subunit	R
cg2678			1,75		ABC-type putative dipeptide/oligopeptide transporter, substrate-binding lipoprotein	E
cg2686	1,79				Putative transcriptional regulator, TetR-family	
cg2687	1,54			*metB*	Cystathionine gamma-synthase	E
cg2695	1,97				ABC-type transporter, ATPase subunit	R
cg2704			0,35		ABC-type putative sugar transporter, permease subunit	G
cg2705			0,44	*amyE*	ABC-type putative sugar transporter, substrate-binding lipoprotein	G
cg2707	2,51		0,40		Conserved hypothetical protein	
cg2708	4,34		0,37	*msiK1*	ABC-type putative sugar transporter, ATPase subunit	G
cg2732		1,86	2,41	*gntV*	Gluconokinase	G
cg2770	1,56				Conserved hypothetical protein	
cg2792	1,79			*nadE*	NAD(+) synthase (glutamine-hydrolyzing)	H
cg2794	2,95				Conserved hypothetical protein	
cg2796	0,10		1,93		Conserved hypothetical protein, MMGE/PRPD-family, putative involved in propoinate catabolism	R
cg2797	0,06				Conserved hypothetical protein	S
cg2800	1,79			*pgm*	Phosphoglucomutase	G
cg2805			0,64	*psp4*	Putative secreted protein	
cg2810			2,91		Putative secondary H+/Na+:glutamate/dicarboxylate symporter, dicarboxylate/amino acid:cation symporter (DAACS) family	C
cg2811			1,58		ABC-type lipoprotein release transporter, permease subunit	V
cg2833			6,77	*cysK*	O-Acetylserine (Thiol)-Lyase	E
cg2835	0,62				Putative acetyltransferase	R
cg2837	2,02			*sucC*	Succinate--CoA ligase (ADP-forming), beta subunit	C
cg2838	1,95				Putative dithiol-disulfide isomerase	Q
cg2840			0,55	*actA*	Putative coenzyme A transferase	C
cg2842	0,56		0,65	*phoU*	Phosphate transport system protein PhoU, putative phosphate uptake regulator	P
cg2846	2,83	3,59	1,58	*pstS*	ABC-type putative phosphate transporter, substrate-binding lipoprotein (TC 3.A.1.7.1)	P
cg2850			1,79		Conserved hypothetical protein	
cg2862	1,55			*purL*	Phosphoribosylformylglycinamidine synthase subunit	F
cg2863	1,93			*purQ*	Phosphoribosylformylglycinamidine synthase subunit	F
cg2865	2,03			*purS*	Phosphoribosylformylglycinamidine synthase subunit	F
cg2873			1,63	*ptrB*	Oligopeptidase B	E
cg2875	0,53				Hypothetical protein	
cg2880	1,70				Putative hydrolase, HIT-family	FGR
cg2888	4,40			*cgtR3*	Two-component system, transcriptional response regulator	TK

cg2891			1,65	*pqo*	Pyruvate:quinone oxidoreductase	EH
cg2893	1,56				Putative multidrug efflux permease, MFS-type	GEPR
cg2902	1,68				Conserved hypothetical protein, hydrolases of the HAD superfamily	R
cg2909	0,66			*otsB*	Trehalose phosphatase	G
cg2911	0,48				ABC-type putative Mn/Zn transporter, substrate-binding lipoprotein	P
cg2924			0,65	*cysS*	Cysteine--tRNA ligase	J
cg2925	0,47		0,55	*ptsS*	Phosphotransferase system (PTS), sucrose-specific enzyme IIBCA component	G
cg2939	1,83				ABC-type putative dipeptide/oligopeptide transporter, ATPase subunit	EP
cg2942		0,58			Putative transcriptional regulator, AsnC-family	K
cg2943		1,59			Putative membrane protein	
cg2949	1,55				Putative secreted protein	
cg2956			0,65		Putative secreted protein	
cg2958			2,08	*butA*	L-2,3-Butanediol dehydrogenase/acetoin reductase	QR
cg2963		2,22		*clpC*	Putative ATP-dependent protease (heat shock protein)	O
cg2969			1,55		Hypothetical protein, similar to acyl-CoA synthetases (AMP-forming)/AMP-acid ligases	
cg2977		0,54			Putative membrane protein	
cg3000	0,36				Putative thiosulfate sulfurtransferase	P
cg3007	0,50				Conserved hypothetical protein	S
cg3008		1,64	1,70	*porA*	Porin	
cg3009			2,49	*porH*	Porin, cation-specific	
cg3021			1,62		Putative peptidase M20/M25/M40 family	E
cg3043	2,01				Putative NTP pyrophosphohydrolase/oxidative damage repair enzyme	LR
cg3048	0,59			*pta*	Phosphate acetyltransferase	C
cg3049	1,69			*fpr1*	Ferredoxin--NADP(+) reductase	ER
cg3058		0,57	0,40	*tnp8b(ISCg8a)*	Transposase	
cg3068	1,79			*fda*	Fructose-bisphosphate aldolase	G
cg3078	1,52				Hypothetical protein	
cg3079	1,89			*clpB*	Putative ATP-dependent protease (heat shock protein)	O
cg3097	2,17			*hspR*	Putative transcriptional regulator, MerR-family	K
cg3099	1,82		1,86	*grpE*	Chaperone GrpE, heat shock protein	O
cg3100		0,56	2,76	*dnaK*	Chaperone DnaK, heat shock protein	O
cg3107	1,70			*adhA*	Alcohol dehydrogenase	R
cg3112			2,06	*cysZ*	Sulfate permease	R
cg3113			2,96	*cysY*	Sirohydrochlorin ferrochelatase	S
cg3114			2,90	*cysN*	Sulfate adenylyltransferase subunit 1	P
cg3115			2,54	*cysD*	Sulfate adenylyltransferase subunit 2	EH
cg3116			2,55	*cysH*	Adenosine phosphosulfate reductase	EH
cg3118			3,48	*cysI*	Ferredoxin-sulfite reductase	P
cg3119			2,36	*fpr2*	Ferredoxin--NADP(+) reductase	ER
cg3120			1,62		Conserved hypothetical protein	S
cg3138	1,64				Putative membrane protease subunit, stomatin/prohibitin homolog	O
cg3140		0,13	0,35	*tagA1*	DNA-3-methyladenine glycosylase	L

					I	
cg3141	1,54	0,19	0,46		Hypothetical protein	C
cg3142	2,33				Putative membrane protein	
cg3143	1,87				Conserved putative secreted protein	R
cg3156	1,73				Putative secreted protein	
cg3157			1,55		Putative secreted protein	V
cg3159	1,57				Putative universal stress protein UspA or related nucleotide-binding protein	T
cg3169	1,86		1,91	*pck*	Phosphoenolpyruvate carboxykinase (GTP)	C
cg3175			0,50		Putative membrane protein	S
cg3179	0,64			*fadD2*	Putative long-chain-fatty-acid--CoA ligase	IQ
cg3186	0,57			*cmt2*	Trehalose corynomycolyl transferase	R
cg3192			2,16		Putative secreted or membrane protein	
cg3193			1,96		Putative membrane-associated phospholipid phosphatase	I
cg3195	2,09				Putative flavin-containing monooxygenase	P
cg3196			0,60	*glf*	UDP-galactopyranose mutase	M
cg3197			0,63	*psp5*	Putative secreted protein	
cg3200	2,02				Putative acyltransferase family	I
cg3201	1,74			*serS*	Serine--tRNA ligase	J
cg3202	4,03				Putative transcriptional regulator, GntR-family	K
cg3211	7,30				Putative secreted protein	
cg3212	1,88				Putative carboxymuconolactone decarboxylase subunit	S
cg3213	2,82				Putative secreted protein	
cg3218			1,94		Pyruvate kinase	G
cg3219	1,91	1,71		*ldh*	L-Lactate dehydrogenase	C
cg3223			1,54		Conserved hypothetical protein, putative FMN reductase	R
cg3226			0,56		Putative MFS-type L-lactate permease	
cg3227	2,25		0,56	*lldA*	Quinone dependent L-lactate dehydrogenase	C
cg3231		0,59			Hypothetical protein	
cg3233	1,72				Hypothetical protein	S
cg3234	1,81				Putative metal-dependent amidase/aminoacylase/carboxypeptidase	R
cg3236	2,14	1,83		*msrA*	Protein-methionine-S-oxide reductase	O
cg3237		1,68	2,57	*sod*	Superoxide dismutase	P
cg3240		2,25	3,44		Putative multidrug efflux permease, MFS-type	
cg3253	1,56			*mcbR*	Global transcriptional repressor of sulfur metabolism, TetR-family	
cg3254	0,58				Putative membrane protein	S
cg3255	7,35	1,64		*uspA3*	Universal stress protein E	T
cg3264	1,72				Conserved hypothetical protein	KT
cg3265			1,60		Hypothetical protein	
cg3272			0,57		Putative membrane protein	S
cg3275	1,66			*fdxA*	Putative ferredoxin	C
cg3277			0,64		Hypothetical protein, containing double-stranded beta-helix domain	S
cg3282	2,30				Putative Cu2+ transporting P-type ATPase	P
cg3292			0,56		Putative heavy-metal ion transporting P-type ATPase	P
cg3300			0,52		Putative Cu2+ transporting P-type ATPase	P
cg3301			0,63		Putative sugar/metabolite permease, MFS-type	

cg3303			0,43		Putative transcriptional regulator, PadR-family	K
cg3304	1,78			*dnaB*	Putative replicative DNA helicase	L
cg3306		0,54	0,41	*rplI*	50S ribosomal protein L9	J
cg3307		0,34	0,42	*ssb*	Single-strand binding protein	L
cg3308	1,53	0,63		*rpsF*	30S ribosomal protein S6	J
cg3315	0,45		2,20		Putative transcriptional regulator, MarR-family	K
cg3316	0,46		2,05		Putative universal stress protein or related nucleotide-binding protein	T
cg3317	1,69				Putative membrane protein	
cg3319	2,46				Conserved hypothetical protein	R
cg3323	3,69				Inositol-3-phosphate synthase	I
cg3327		2,94	2,89	*dps*	Putative starvation-induced DNA protecting protein	P
cg3337	1,79				Putative membrane protein	V
cg3338	3,33				Putative membrane protein	V
cg3340			1,64	*dadA*	D-Amino-acid dehydrogenase	E
cg3343	2,16		1,67		Putative secreted membrane protein	
cg3344	2,29				Putative nitroreductase	C
cg3346	2,08			*leuS*	Leucine--tRNA ligase	J
cg3357			0,47	*trpP*	Permease, tryptophan-specific	
cg3359		0,62		*trpE*	Anthranilate synthase subunit I	EH
cg3359			0,39	*trpE*	Anthranilate synthase subunit I	EH
cg3369	2,08			*qcrA2*	Putative rieske iron-sulfur protein	PR
cg3370		0,58			Putative NADH-dependent flavin oxidoreductase	C
cg3380	0,61				Putative oxidoreductase protein	
cg3423	1,68			*trxC*	Thioredoxin	OC
cg3432	0,48			*rpmH*	50S ribosomal protein L34	J

a Locus tag according to [50]

b COG class according to [11]
